# Ecological Guild Evolution and the Discovery of the World's Smallest Vertebrate

**DOI:** 10.1371/journal.pone.0029797

**Published:** 2012-01-11

**Authors:** Eric N. Rittmeyer, Allen Allison, Michael C. Gründler, Derrick K. Thompson, Christopher C. Austin

**Affiliations:** 1 Department of Biological Sciences and Museum of Natural Science, Louisiana State University, Baton Rouge, Louisiana, United States of America; 2 Bishop Museum, Honolulu, Hawaii, United States of America; 3 Department of Ecology and Evolutionary Biology, Cornell University, Ithaca, New York, United States of America; University of Arkanas, United States of America

## Abstract

Living vertebrates vary drastically in body size, yet few taxa reach the extremely minute size of some frogs and teleost fish. Here we describe two new species of diminutive terrestrial frogs from the megadiverse hotspot island of New Guinea, one of which represents the smallest known vertebrate species, attaining an average body size of only 7.7 mm. Both new species are members of the recently described genus *Paedophryne*, the four species of which are all among the ten smallest known frog species, making *Paedophryne* the most diminutive genus of anurans. This discovery highlights intriguing ecological similarities among the numerous independent origins of diminutive anurans, suggesting that minute frogs are not mere oddities, but represent a previously unrecognized ecological guild.

## Introduction

Living vertebrates range in size over 3,000 fold. The breadth and limits on vertebrate size have been of great interest to biologists due to the functional and physiological constraints associated with extreme body size. The largest extant vertebrate is the blue whale (*Balaenoptera musculus*, average adult size 25.8 m) [1] while the smallest is a fish (*Paedocypris progenetica*, adult size 7.9–10.3 mm) [2]. Both species are aquatic and biologists have speculated that the buoyancy of water may play a role in facilitating the evolution of both large and small size [3]–[5]. Extreme miniaturization, however, has evolved independently at least eleven times in terrestrial frogs. Here we describe two new species of diminutive terrestrial frogs from the island of New Guinea, one of which represents the smallest known vertebrate species, attaining an average body size of only 7.7 mm (range 7.0–8.0 mm). We identify ecological similarities among the most diminutive frog species suggesting that the independent origins of minute frogs are not merely evolutionary outliers, but represent a previously undocumented ecological guild found in moist leaf litter of tropical wet-forests.

## Results

### Taxonomic treatment

Amphibia, Linnaeus, 1758

Anura, Rafinesque, 1815

Microhylidae, Günther, 1858

Asterophryinae, Günther, 1858

*Paedophryne*, Kraus 2010

*Paedophryne amauensis*, sp. nov. (urn:lsid:zoobank.org:act:496F26AB-CD82-4A9C-944C-070EC86ADAA4)

#### Etymology

The species epithet refers to the type locality, near Amau Village, Central Province, Papua New Guinea.

#### Holotype

LSUMZ 95000 (field tag CCA 5739), adult male, collected by C.C. Austin and E.N. Rittmeyer near Amau Village, Central Province, Papua New Guinea, 09.9824°S, 148.5785°E, 177 m, 7 August 2009.

#### Paratypes

LSUMZ 95001, same data as holotype, except collected 6 August 2009; LSUMZ 95002, same data as holotype, except collected 10 August 2009; LSUMZ 95003-4, same data as holotype, except collected 12 August 2009; LSUMZ 95005-6, same data as holotype, except collected 14 August 2009.

#### Diagnosis

**Figure 1:**
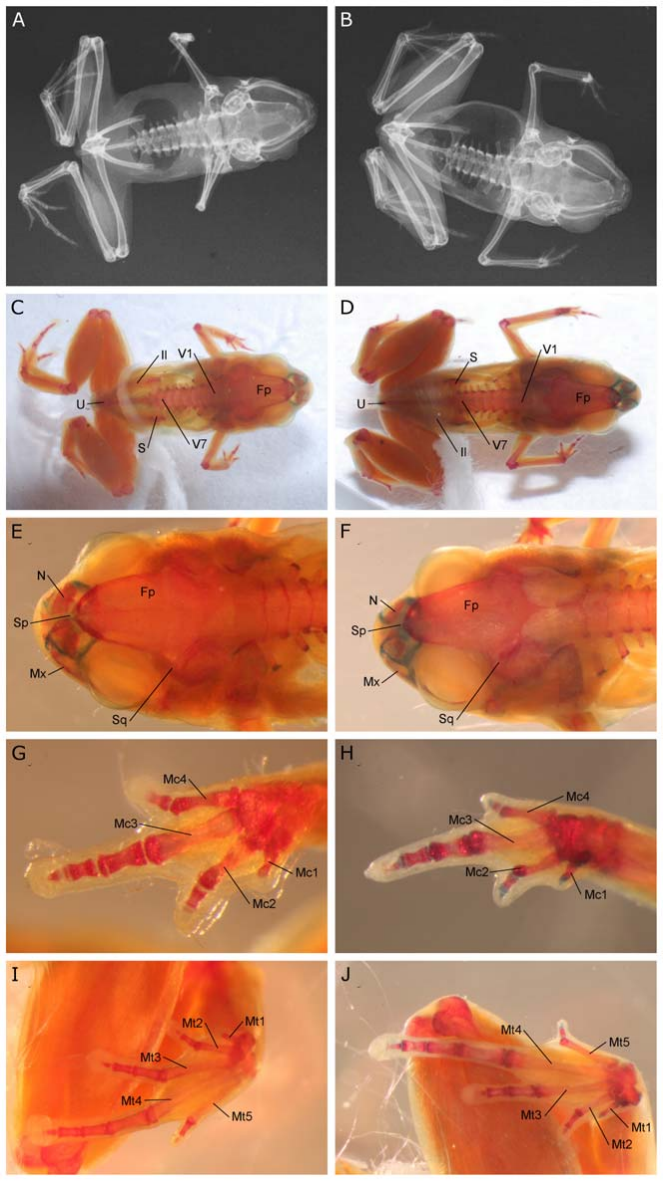
Osteological characters of *Paedophryne amauensis*, *P. swiftorum*. **A.** X-ray of paratype of *Paedophryne amauensis* (LSUMZ 95002). **B.** X-ray of paratype of *P. swiftorum* (BPBM 31886). **C,E,G,I.** Photos of cleared and double-stained paratype of *P. amauensis* (LSUMZ 95002). **C.** Whole body. **E.** Head. **G.** Hand. **I.** Foot. **D,F,H,J.** Photos of cleared and double-stained paratype of *P. swiftorum* (BPBM 31886). **D.** Whole body. **F.** Head. **H.** Hand. **J.** Foot. Skeletal elements labeled as follows: Fp, frontoparietal; Il, illium; Mc1-4, metacarpals 1-4; Mt1-5, metatarsals 1-5; Mx, maxilla; N, nasal; S, Sacrum; Sp, sphenethemoid; Sq, squamosal; U, urostyle; V1, first presacral vertebra; V7, seventh presacral vertebra.

A minute microhylid (male SVL = 7.0–8.0 mm) of the genus *Paedophryne* based on the following combination of characters: eleutherognathine jaw, 7 presacral vertebrae, first digits of hand and foot reduced to single elements, prepollex and prehallux reduced to single elements (Fig. 1). Legs moderately long (TL/SVL = 0.478–0.507), snout broad and short (EN/SV = 0.075–0.084, EN/IN = 0.667–0.765), and eye relatively large (EY/SVL = 0.127–0.150). Digits un-webbed with slightly enlarged discs (3F/SVL = 0.025–0.033; 4T/SVL = 0.036–0.050). First finger and first toe reduced to vestigial nubs, second and fourth fingers and second and fifth toes also markedly reduced. Dorsal coloration dark brown with irregular tan to rusty-brown blotches; lateral and ventral surfaces dark brown to slate grey with irregular bluish-white speckling. Detailed mensural characters and proportions provided in Table 1 and Table 2.

**Table 1:** Mensural characters of *Paedophryne amauensis* and *P. swiftorum*.

Catalogue No.	Species	Sex	SVL	TL	EY	EN	IN	SN	HW	HL	3F	4T
LSUMZ 95000*	*P. amauensis*	Male	7.50	3.80	1.05	0.60	0.80	0.85	2.85	2.15	0.25	0.30
LSUMZ 95001	*P. amauensis*	Male	7.00	3.55	0.95	0.55	0.75	0.65	2.75	1.90	0.20	0.25
LSUMZ 95002	*P. amauensis*	Male	7.85	3.75	1.00	0.60	0.80	0.95	2.75	2.25	0.20	0.30
LSUMZ 95003	*P. amauensis*	Male	8.00	3.90	1.20	0.60	0.85	0.75	2.90	2.30	0.25	0.40
LSUMZ 95004	*P. amauensis*	Male	8.00	3.95	1.10	0.60	0.90	0.95	2.90	2.20	0.25	0.30
LSUMZ 95005	*P. amauensis*	Male	7.70	3.80	1.00	0.65	0.85	0.95	2.90	2.10	0.20	0.30
LSUMZ 95006	*P. amauensis*	Male	7.85	3.80	1.10	0.60	0.85	0.85	2.75	2.25	0.20	0.30
BPBM 31880	*P. swiftorum*	Male	8.50	3.95	1.20	0.55	0.95	0.80	2.80	2.40	0.15	0.35
BPBM 31881	*P. swiftorum*	Male	8.90	3.80	1.25	0.60	0.95	0.85	2.90	2.50	0.20	0.40
BPBM 31882	*P. swiftorum*	Male	8.40	3.70	1.25	0.60	0.95	0.80	3.00	2.40	0.20	0.35
BPBM 31883*	*P. swiftorum*	Male	8.55	4.00	1.25	0.55	0.95	0.85	3.00	2.50	0.15	0.35
BPBM 31884	*P. swiftorum*	Male	8.25	3.85	1.15	0.55	0.90	0.80	2.90	2.35	0.20	0.35
BPBM 31885	*P. swiftorum*	Juvenile	4.45	1.75	0.75	0.25	0.50	0.50	1.75	1.40	0.20	0.20
BPBM 31886	*P. swiftorum*	Male	8.50	4.00	1.25	0.55	0.90	0.85	3.00	2.45	0.20	0.40

Mensural data for *Paedophryne amauensis* sp. nov. and *P. swiftorum* sp. nov. Measurements, terminology, and abbreviations follow Kraus [13]: body length (SVL), tibia length (TL), horizontal eye diameter (EY), distance from anterior of eye to naris (EN), internarial distance between external nares (IN), distance from anterior of eye to tip of snout (SN), head width at center of tympana (HW), head length from posterior of tympana to tip of snout (HL), width of third finger disc (3F), and width of fourth toe disc (4T). All measurements were made to the nearest 0.05 mm using dial calipers or an optical micrometer. Asterisks (*) indicate holotypes.

**Table 2:** Relevant proportions of *Paedophryne* species.

Cat. No.	Species	Sex	SVL	TL/SV	EN/SV	IN/SV	SN/SV	EY/SV	HW/SV	HL/SV	3F/SV	4T/SV	EN/IN	3F/4T	HL/HW	EY/SN
LSUMZ 95000*	*P. amauensis*	Male	7.50	0.507	0.080	0.107	0.113	0.140	0.380	0.287	0.033	0.040	0.750	0.833	0.754	1.235
LSUMZ 95001	*P. amauensis*	Male	7.00	0.507	0.079	0.107	0.093	0.136	0.393	0.271	0.029	0.036	0.733	0.800	0.691	1.462
LSUMZ 95002	*P. amauensis*	Male	7.85	0.478	0.076	0.102	0.121	0.127	0.350	0.287	0.025	0.038	0.750	0.667	0.818	1.053
LSUMZ 95003	*P. amauensis*	Male	8.00	0.488	0.075	0.106	0.094	0.150	0.363	0.288	0.031	0.050	0.706	0.625	0.793	1.600
LSUMZ 95004	*P. amauensis*	Male	8.00	0.494	0.075	0.113	0.119	0.138	0.363	0.275	0.031	0.038	0.667	0.833	0.759	1.158
LSUMZ 95005	*P. amauensis*	Male	7.70	0.494	0.084	0.110	0.123	0.130	0.377	0.273	0.026	0.039	0.765	0.667	0.724	1.053
LSUMZ 95006	*P. amauensis*	Male	7.85	0.484	0.076	0.108	0.108	0.140	0.350	0.287	0.025	0.038	0.706	0.667	0.818	1.294
BPBM 31880	*P. swiftorum*	Male	8.50	0.465	0.065	0.112	0.094	0.141	0.329	0.282	0.018	0.041	0.579	0.429	0.857	1.500
BPBM 31881	*P. swiftorum*	Male	8.90	0.427	0.067	0.107	0.096	0.140	0.326	0.281	0.022	0.045	0.632	0.500	0.862	1.471
BPBM 31882	*P. swiftorum*	Male	8.40	0.440	0.071	0.113	0.095	0.149	0.357	0.286	0.024	0.042	0.632	0.571	0.800	1.563
BPBM 31883*	*P. swiftorum*	Male	8.55	0.468	0.064	0.111	0.099	0.146	0.351	0.292	0.018	0.041	0.579	0.429	0.833	1.471
BPBM 31884	*P. swiftorum*	Male	8.25	0.467	0.067	0.109	0.097	0.139	0.352	0.285	0.024	0.042	0.611	0.571	0.810	1.438
BPBM 31886	*P. swiftorum*	Male	8.50	0.471	0.065	0.106	0.100	0.147	0.353	0.288	0.024	0.047	0.611	0.500	0.817	1.471
BPBM 17975	*P. kathismaphlox*	Female	10.40	0.35	0.067	0.087	0.13	0.12	0.38	0.28	0.024	0.037	0.78	0.66	0.74	0.92
BPBM 17976	*P. kathismaphlox*	Female	10.90	0.38	0.073	0.092	0.12	0.12	0.35	0.32	0.028	0.032	0.80	0.86	0.92	1.00
BPBM 17977*	*P. kathismaphlox*	Female	10.50	0.39	0.076	0.095	0.13	0.12	0.35	0.31	0.031	0.037	0.80	0.85	0.89	0.93
BPBM 35353	*P. kathismaphlox*	Male	10.10	0.39	0.079	0.099	0.12	0.12	0.37	0.31	0.029	0.035	0.80	0.83	0.84	1.00
BPBM 16433*	*P. oyatabu*	Female	11.30	0.398	0.062	0.097	0.124	0.133	0.372	0.319	0.025	0.031	0.636	0.800	0.857	1.071

Relevant proportions of *P. amauensis* sp. nov., *P. swiftorum* sp. nov., and the two previously described species of *Paedophryne*. Values for *P. kathismaphlox* and *P. oyatabu* from Kraus [13]. Asterisks indicate holotypes.

*Paedophryne amanuensis* is distinguished from all congeners by its smaller size (SVL = 10.1–10.9 mm in *P. kathismaphlox*, 11.3 mm in *P. oyatabu*, 8.3–8.9 mm in *P. swiftorum*) and longer legs (TL/SVL = 0.35–0.39 in *P. kathismaphlox*, 0.40 in *P. oyatabu*, 0.427–0.471 in *P. swiftorum*). *Paedophryne amauensis* is further distinguished from *P. oyatabu* and *P. swiftorum* by its longer, narrower head (EN/SV = 0.062, EN/IN = 0.64 in *P. oyatabu*; EN/SV = 0.064–0.071, EN/IN = 0.579–0.632 in *P. swiftorum*), and from *P. kathismaphlox* by its shorter, broader head (EN/SV = 0.067–0.079; EN/IN = 0.78–0.80 in *P. kathismaphlox*). The call of *P. amauensis* differs from that of *P. swiftorum* by its higher dominant frequency (7300 Hz in *P. swiftorum*) and by consisting of single notes, rather than eight paired notes as in *P. swiftorum*. The calls of *P. kathismaphlox* and *P. oyatabu* are unknown.

#### Call

**Figure 2:**
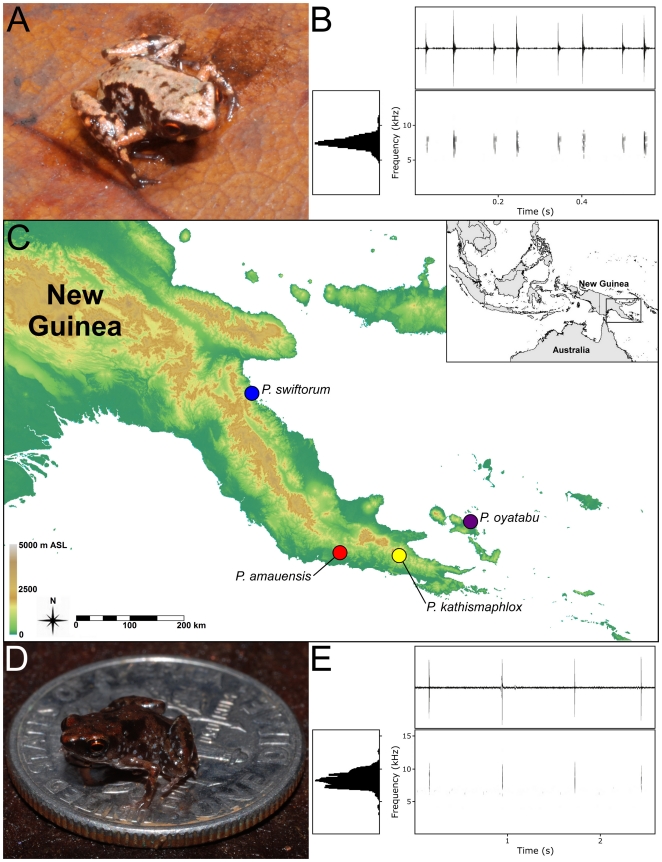
Type localities, call sonograms, and photographs of *Paedophryne* species. **A.** Photograph of paratype of *Paedophryne swiftorum* in life (BPBM 31880). **B.** Waveform (upper right), power spectrum (lower left) and spectrogram (lower right) of a single call series consisting of four double notes of the holotype of *P. swiftorum* (BPBM 31883). **C.** Type localities of the four species of *Paedophryne*. Blue: *P. swiftorum*; red: *P. amauensis*; yellow: *P. kathismaphlox*; purple: *P. oyatabu*. **D.** Photograph of paratype of *P. amanuensis* (LSUMZ 95004) on U.S. dime (diameter 17.91 mm). **E.** Waveform (upper right), power spectrum (lower left) and spectrogram (lower right) of the first four notes of the call of the holotype of *P. amauensis* (LSUMZ 95000).

This species is crepuscular and calls from within leaf litter in primary forest at dawn and dusk. Its call consists of a continuous series of high-pitched notes with a dominant frequency of ∼8400–9400 Hz. Individual notes range in duration from 2–14 ms and are produced at a rate of 1.5 notes/s (Fig. 2; Table 3). The overall acoustic impression is that of a stridulating insect. Individuals generally call from one to three minutes and then rest briefly before resuming. In a 5.5 minute recorded sequence, one individual (NS2, Table 3) produced a total of 355 calls in four groups, with the interval between groups ranging from 3.3 to 40.8 s.

**Table 3:** Call characters of *Paedophryne amauensis*.

Specimen	Total Calls Recorded	Mean Call Note Duration (s)	Range in Call Note Duration (s)	Mean Internote Duration (s)	Range in Internote Duration (s)	Calling Frequency (calls/s)	Dominant Frequency (mHz)
LSUMZ 95000*	139	0.0055	0.0025–0.0102	0.624	0.1843–0.7875	1.59	9200
LSUMZ 95004	86	0.005	0.0029–0.0084	0.7056	0.5889–0.8617	1.44	8820
NS 1	252	0.0055	0.0030–0.0092	0.6934	0.5684–1.098	1.43	8440
NS 2	355	0.0051	0.0021–0.0142	0.6727	0.5533–1.0980	1.54	8440

Call characters of *Paedophryne amauensis*. NS refers to specimens not collected. Asterisk indicates the holotype.

*Paedophryne swiftorum*, sp. nov. (urn:lsid:zoobank.org:act:6F724864-05A5-4729-AB27-7093A64F90F2)

#### Etymology

The species epithet honors the Swift family, in recognition of their generous contributions that enabled the establishment of the Kamiali Biological Station, where the type series was collected.

#### Holotype

BPBM 31883 (field tag AA 19195), adult male, collected by A. Allison, M.C. Gründler, E.N. Rittmeyer, and D.K. Thompson at Kamiali Wildlife Management Area, 1.3 km N, 6.2 km W of Cape Dinga, Cliffside Camp, Morobe Province, Papua New Guinea, 07.255997°S, 147.092879°E, 500 m elevation, 14 July 2008.

#### Paratypes

BPBM 31879, same data as holotype, except collected 8 July 2008; BPBM 31880, same data as holotype, except collected 10 July 2008; BPBM 31881-82, same data as holotype, except collected 11 July 2008; BPBM 31884 collected by M. Gründler at Kamiali Wildlife Management Area, Pinetree Camp, Morobe Province, Papua New Guinea, 07.257906°S, 147.06335°E, 950 m elevation, 12 July 2008; BPBM 31885, same data as BPBM 31884, except an unsexed juvenile collected on 13 July 2008; BPBM 31886, same data as holotype, except collected 13 July 2008.

#### Diagnosis

A minute microhylid (SVL = 8.25–8.90 mm) of the genus *Paedophryne* based on the following combination of characters: eleutherognathine jaw, 7 presacral vertebrae, first digits of hand and foot reduced to single elements, prepollex and prehallux reduced to single elements (Fig. 1). Legs moderately long (TL/SVL = 0.427–0.471), snout short and broad (EN/SV = 0.064–0.071; EN/IN = 0.579–0.623), and eyes relatively large (EY/SVL = 0.139–0.149). Fingers lacking enlarged discs (3F/SVL = 0.018–0.024), toes with slightly enlarged discs (4T/SVL = 0.041–0.047). Digits un-webbed; first finger and first toe reduced to vestigial nubs, second and fourth fingers and second and fifth toes also markedly reduced. Dorsum dark brown with irregular tan to rusty brown blotches or a broad tan mid-dorsal stripe; chin and throat dark brown, abdomen lighter brown, occasionally mottled with tan. Detailed mensural characters and proportions provided in Table 1 and Table 2.

*Paedophryne swiftorum* is distinguished from *P. oyatabu* and *P. kathismaphlox* by its smaller size (SVL = 10.1–10.9 mm in *P. kathismaphlox*, 11.3 mm in *P. oyatabu*), longer legs (TL/SVL = 0.35–0.39 in *P. kathismaphlox*, 0.40 in *P. oyatabu*,), and larger eyes (EY/SV = 0.12 in *P. kathismaphlox*, 0.13 in *P. oyatabu*). *Paedophryne swiftorum* is further distinguished from *P. kathismaphlox* by its broader head (EN/IN = 0.78–0.80 in *P. kathismaphlox*). It is distinguished from *P. amauensis* by its larger size (SVL = 7.0–8.0 mm in *P. amauensis*), shorter legs (TL/SVL = 0.478–0.507 in *P. amauensis*), and shorter, broader head (EN/SV = 0.075–0.084; EN/IN = 0.667–0.765 in *P. amauensis*). The call of *P. swiftorum* differs from that of *P. amauensis* by its lower dominant frequency (8400–9400 Hz in *P. amauensis*), and by consisting of a series of four double notes, rather than repeated single notes as in *P. amauensis*. The individual notes are otherwise similar to *P. amauensis*. The calls of other *Paedophryne* species are unknown.

#### Call

The calling ecology of *Paedophryne swiftorum* is similar to that of *P. amauensis* – it is generally crepuscular; however, it calls diurnally during particularly wet conditions. It does not call nocturnally regardless of rainfall. The call generally consists of four double notes (Fig. 2; Table 4) delivered in a continuous series at the rate of 0.66 calls/s. Each note is around 7 ms in duration and the entire call lasts approximately 0.5 seconds. The interval between notes is 40–50 ms within a double note series and 85–100 ms between each double note series. The dominant frequency averages 7300 Hz. Some individuals occasionally produce calls of only six notes, invariably consisting of double notes, and otherwise similar to eight-note calls. The acoustic characteristics of the call and the tendency of males to call continuously within a chorus produces an uncanny resemblance to stridulating orthopteran insects.

**Table 4:** Call characters of *Paedophryne swiftorum*.

Specimen	Total Calls Recorded	Mean Call Duration (s)	Notes per Call (mode)	Range in Call Duration (s)	Mean Inter-Call Duration (s)	Range in Inter-Call Duration (s)	Calling Frequency (calls/s)	Dominant Frequency (mHz)
BPBM 31881	71	0.494	8	0.3552–0.5588	1.0174	0.8400–1.7620	0.66	7220
BPBM 31883*	20	0.5589	8	0.5066–0.6857	0.9888	0.8607–1.2433	0.65	7400

Call characters of *Paedophryne swiftorum*. Asterisk indicates the holotype.

### Morphology

Most miniaturized species show an overall reduction and simplification of their bauplan [6]. Miniaturized anurans in particular often show a reduced number of digits and phalangeal elements [7], [8], and the loss or reduction of some cranial elements [8], [9]. The four known *Paedophryne* species corroborate the trend of digital reduction: multiple digits are reduced in size and the first digits of the hand and foot are reduced to miniscule nubs. Further, like many other miniaturized anurans, *Paedophryne* exhibit reduced numbers of phalangeal elements (Fig. 1): all species have phalangeal formulas on the manus of 1-2-3-2 (as opposed to the typical 2-2-3-3 [10]), and on the pes of 1-2-3-4-2 (as opposed to the typical 2-2-3-4-3 [10]). The skull of *Paedophryne* is largely ossified, though several elements, particularly those more anterior, are reduced in size (e.g. nasals) or at least partially chondrified (e.g. sphenethmoid). Several elements that typically ossify late in anuran development (e.g. columella, mentomeckelian) are present and partially or entirely ossified, whereas others (e.g. sphenethmoid) are chondrified (Fig. 1). This pattern may suggest developmental truncation as a mechanism for the extremely reduced body size of *Paedophryne*, as has been proposed for other miniaturized anurans [9]; however, little is known of the cranial ontogeny in direct-developing anurans. Direct development has evolved numerous times independently and cranial ontogeny has only been examined in detail in a small number of species. These examined species show varied sequences of ossification. Some (e.g. *Philautus silus*) show patterns similar to typical anurans in which cranial elements involved in the braincase ossify early in development and those associated with the adult jaw ossify later [11], whereas others (e.g. *Eleutherodactylus coqui*) show drastically different patterns in which cranial elements associated with the adult jaw ossify early in development [12]. The cranial ontogeny has not been examined in any asterophryine frogs, which represent an independent origin of direct development from any examined species, thus it is not clear from the patterns of cranial ossification if the diminutive size of *Paedophryne* is the result of developmental truncation (as has been hypothesized for many other minute frog species [6], [9], proportional dwarfism, or some combination of these or other mechanisms. In addition to these patterns of digital and cranial reduction, *Paedophryne* show a reduction in the number of presacral vertebrae (7 in *Paedophryne*, Fig. 1, versus 8 in most other anurans and other asterophryines [10], [13], and an overall rather juvenile appearance.

## Discussion

Miniaturization, the reduction in body size necessitating drastic alterations to an organism's physiology, ecology, and behavior, is known from every major vertebrate lineage and nearly all major groups of animals [6]. Yet among vertebrates only teleost fishes approach the extreme size of *Paedophryne amauensis*; the smallest known actinopterygian fish is *Paedocypris progenetica*, maturing at 7.9 mm [2], whereas the smallest known vertebrate excluding teleosts and anurans is a gecko (*Spherodactylus ariasae*, mean SVL = 16.3 mm) [14] or a salamander (*Thorius arboreus*, mean SVL = 17.0 mm) [15]. Miniaturization has occurred repeatedly in anurans: the 29 smallest species (maximum male SVL<13 mm) include representatives from 5 families and 11 genera (Table 5) [7], [13], [16]–[24]. Several large frog families (e.g. Bufonidae, Hylidae, Ranidae) lack extremely miniaturized species, whereas other families include numerous minute taxa: 15 of these species are microhylids, including representatives of 7 genera. This distribution of miniaturization among frog families suggests that the evolution of miniaturization has been nonrandom with respect to phylogeny.

**Table 5:** Sizes and mode of reproduction in extremely miniaturized frogs.

Genus	Species	Family: Subfamily	Male Max. SVL	Female Max. SVL	Male Min. SVL	Male Mean SVL	Female Min. SVL	Female Mean SVL	Mode of Reproduction	Reference
***Paedophryne***	***amauensis***	**Microhylidae: Asterophryinae**	**8**	—	**7**	**7.7**	—	—	**Direct**	**This Study**
***Paedophryne***	***swiftorum***	**Microhylidae: Asterophryinae**	**8.9**	—	**8.25**	**8.5**	—	—	**Direct**	**This Study**
*Brachycephalus*	*didactylus*	Brachycephalidae	9	—	8	8.6	—	10.2	Direct	[7], [16], [17], [55]
*Syncope*	*carvalhoi*	Microhylidae: Gastrophryninae	9.6	11.7	—	—	10.9	—	Direct?	[18], [56]
*Eleutherodactylus*	*iberia*	Eleutherodactylidae: Eleutherodactylinae	10	10.5	9.6	9.8	—	10.5	Direct	[7], [16], [17]
*Brachycephalus*	*hermogenesi*	Brachycephalidae	—	10.5	—	—	—	10.5	Direct	[16], [57]
*Paedophryne*	*kathismaphlox*	Microhylidae: Asterophryinae	10.1	10.9	10.1	—	10.4	—	Direct	[13]
*Sechellophryne*	*gardineri*	Sooglossidae	—	—	—	10.2	—	11.5	Direct	[7], [16], [58]
*Syncope*	*tridactyla*	Microhylidae: Gastrophryninae	10.3	11.3	—	—	11.2	—	Unknown	[59]
*Paedophryne*	*oyatabu*	Microhylidae: Asterophryinae	—	11.3	—	—	11.3	—	Direct	[13]
*Stumpffia*	*tridactyla*	Microhylidae: Cophylinae	11	—	10	—	—	—	Larval*	[7], [16], [17], [60]
*Aphantophryne*	*minuta*	Microhylidae: Asterophryinae	—	11.8	—	—	—	—	Direct	[20]
*Microhyla*	*supracilius*	Microhylidae: Microhylinae	—	11.8	—	—	—	—	Larval	[21]
*Noblella*	*pygmaea*	Strambomantidae: Holoadeninae	11.1	12.4	10.3	10.7	11.3	—	Direct	[16], [17]
*Syncope*	*antenori*	Microhylidae: Gastrophryninae	11.2	13.2	—	—	12.3	—	Larval*	[56], [60]
*Brachycephalus*	*brunneus*	Brachycephalidae	11.3	12	9.3	10.2	10.9	11.7	Direct	[16], [61]
*Oreophryne*	*minuta*	Microhylidae: Asterophryinae	11.5	—	9.2	—	—	—	Direct	[22]
*Eleutherodactylus*	*orientalis*	Eleutherodactylidae: Eleutherodactylinae	11.5	12	10.7	11	11.2	11.6	Direct	[16], [17]
*Choerophryne*	*allisoni*	Microhylidae: Asterophryinae	11.6	—	11.6	—	—	—	Direct	[23]
*Eleutherodactylus*	*limbatus*	Eleutherodactylidae: Eleutherodactylinae	11.7	11.8	9.8	10.5	11.1	11.6	Direct	[7], [16], [17]
*Noblella*	*myrmecoides*	Strambomantidae: Holoadeninae	—	13.6	—	—	12	—	Direct	[17], [62]
*Brachycephalus*	*nodoterga*	Brachycephalidae	11.8	14.5	—	11.8	12.7	13.4	Direct	[7], [16], [63]
*Stumpffia*	*pygmaea*	Microhylidae: Cophylinae	12	—	10	11	—	12	Larval*	[7], [16], [17], [59]
*Microhyla*	*perparva*	Microhylidae: Microhylinae	12	14.5	10.1	—	11.4	—	Larval	[21], [64]
*Brachycephalus*	*izecksohni*	Brachycephalidae	12.1	13.1	10.3	11.1	12.5	12.8	Direct	[16], [61]
*Eleutherodactylus*	*tetajulia*	Eleutherodactylidae: Eleutherodactylinae	12.3	14	11.6	12	13	13.5	Direct	[16], [65]
*Eleutherodactylus*	*thorectes*	Eleutherodactylidae: Eleutherodactylinae	—	—	—	12.2	—	14.5	Direct	[7], [16], [66]
*Choerophryne*	*burtoni*	Microhylidae: Asterophryinae	12.4	—	12.1	—	—	—	Direct	[24]
*Brachycephalus*	*ferruginus*	Brachycephalidae	12.5	14.5	11.6	12.2	13	13.8	Direct	[16], [17], [67]

Sizes and mode of reproduction of the 29 smallest frog species (i.e. those with a maximum male size <13 mm), arranged by maximum male size, or, if unavailable, mean male size or female size. All values in millimeters (mm). Species described herein are in bold. Direct refers to direct development (i.e. lacking a larval tadpole stage), larval refers to a typical free-swimming, feeding tadpole stage, and larval* refers to a non-feeding larval stage.

Miniaturized animals typically show reduced overall fecundity and increased egg size relative to larger congeners [6]. Of the 29 smallest frogs, 24 (83%) lack a larval tadpole stage and develop directly [7], [13], [16]–[20], [22]–[24], and only two congeners (*Microhyla supracilius*, *M. perparva*) have a typical anuran tadpole stage [21]. These direct developing species belong to clades that include much larger direct developing species, thus direct development may facilitate the evolution of extreme miniaturization in frogs [7]. Miniaturized species also typically express a generally reduced and simplified morphology [6], [8], [9]. These changes are also apparent in *Paedophryne*, which exhibit a reduced number of presacral vertebrae, reduced ossification of several cranial elements, and phalangeal and digital reduction on both the hand and foot (Fig. 1).

All but two species of extremely miniaturized frogs inhabit tropical wet-forest leaf litter; the two exceptions (*Choerophryne burtoni*, *Oreophryne minuta*) inhabit dense moist moss. Frogs are sensitive to water loss [25]–[27] and small species, which have a high surface to volume ratio, are particularly susceptible to desiccation [28]. Indeed, one of smallest known amniote species (*Sphaerodactylus parthenopion*) loses water at much higher relative rates than larger congeners, and is known to select moist microhabitats to compensate [28]. A disproportionate number of tropical wet-forest frogs occur on or near the ground and have life histories dependent on the near constant high moisture content of leaf litter [29]. This may explain the absence of diminutive frogs from temperate forests and tropical dry-forests, where the leaf litter is seasonally dry. Alternatively, the absence of minute frogs from temperate forests may be explained by the evolution of clades including miniaturized species in the wet tropics (i.e. tropical niche conservatism) [30]–[32]; however, this would not explain the apparent absence of these species from tropical dry-forests. Thus, the wet-forest leaf litter may represent an adaptive zone for diminutive frogs. Their small size likely increases their susceptibility to predation by invertebrates [33]–[35], which may account for the absence of diminutive anurans from aquatic habitats, where invertebrate predation is particularly high [33]. This may also explain a tendency for these frogs to inhabit upland regions where invertebrate diversity is less than in the lowlands.

**Figure 3:**
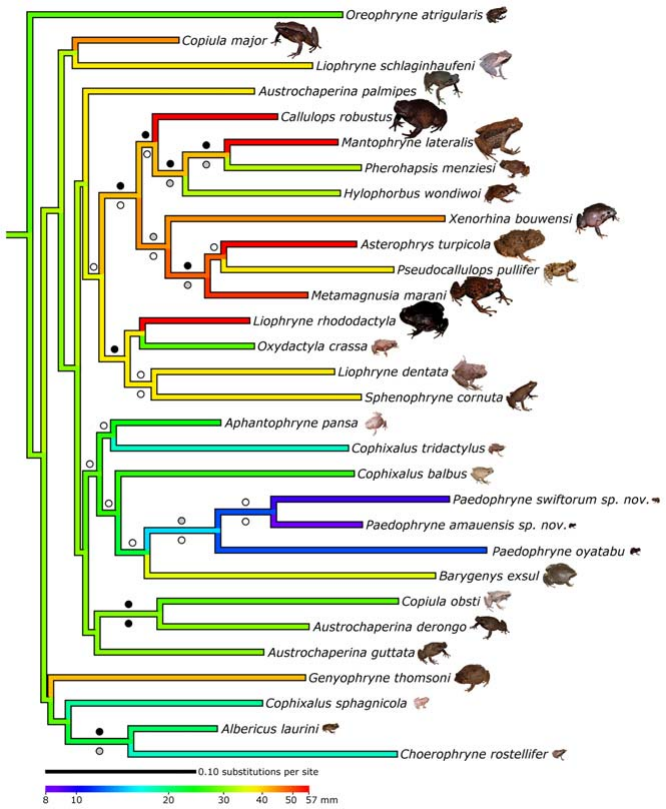
Phylogenetic position of *Paedophryne* and evolution of body size in Asterophryinae. Maximum likelihood phylogeny of *Paedophryne* and asterophryine frogs. Colors of branches correspond to maximum male SVL (*Paedophryne*) or average SVL within each clade on a logarithmic scale (Table 6). Circles above branches correspond to posterior probabilities: black: >0.95; grey: 0.85–0.95; white: 0.5–0.85. Circles below branches correspond to maximum likelihood bootstrap support: black: >95%; grey: 75–95%; white: 50–75%.

Phylogenetic analyses corroborate the monophyly of *Paedophryne* (albeit with moderate support) and suggest a relationship with *Barygenys* and *Cophixalus balbus* (Fig. 3, Fig. S1). Divergences among species within *Paedophryne* are surprisingly deep (mean uncorrected p-distance ≥0.102) and on par with, or greater than, divergences observed among distinct genera of asterophryine frogs (e.g. mean uncorrected p-distance between *Albericus* and *Choerophryne* = 0.11, between *Hylophorbus* and *Mantophryne*+*Pherohapsis* = 0.113). These deep divergences within *Paedophryne* suggest that the extremely diminutive size exhibited by the genus arose early in the radiation of microhylid frogs in New Guinea, thus indicating that these minute anurans have long been a component of the leaf litter community where they occur. Indeed, *Paedophryne amanuensis* and *P. swiftorum* appear to be relatively common inhabitants of leaf litter, judging by the level of calling, and we estimate that calling male *P. swiftorum* are spaced only approximately 50 cm from one another within the leaf litter. Thus, these minute species are likely an important component of the tropical wet-forest ecosystem, both as a predator of small invertebrates such as acarians and collembolans, and as a prey item for larger invertebrates and vertebrates.

The discovery of *Paedophryne amauensis* and *P. swiftorum* also greatly expands the distribution of the genus westward, both north and south of the central mountains. The genus remains restricted to the East Papuan Aggregate Terrain that composes the Papuan Peninsula in eastern New Guinea [36]–[38], supporting Kraus's [13] conclusion on the importance of this geologic entity for the evolution of *Paedophryne*. However, the poorly explored nature of New Guinea and the extremely minute size and atypical, insect-like call of *Paedophryne* species leaves the possibility of a much broader distribution.

These discoveries further reveal intriguing patterns of amphibian diversity in a megadiverse hotspot region and highlight ecological similarities among the most diminutive anurans, suggesting that these species are not merely curiosities, but represent a previously unrecognized ecological guild. Phylogenetic analysis also show genetic divergences among *Paedophryne* species are deep, equal to or greater than among genera of asterophryine frogs, suggesting that the evolution of this miniaturized vertebrate guild arose early in the radiation of New Guinea microhylid frogs. Such discoveries are increasingly critical in this time of global amphibian declines and extinctions.

## Materials and Methods

### Nomenclatural Acts

The electronic version of this document does not represent a published work according to the International Code of Zoological Nomenclature (ICZN), and hence the nomenclatural acts contained in the electronic version are not available under that Code from the electronic edition. Therefore, a separate edition of this document was produced by a method that assures numerous identical and durable copies, and those copies were simultaneously obtainable (from the publication date noted on the first page of this article) for the purpose of providing a public and permanent scientific record, in accordance with Article 8.1 of the Code. The separate print-only edition is available on request from PLoS by sending a request to PLoS ONE, Public Library of Science, 1160 Battery Street, Suite 100, San Francisco, CA 94111, USA along with a check for $10 (to cover printing and postage) payable to “Public Library of Science”. This article has also been digitally archived in the PubMedCentral (www.ncbi.nlm.nih.gov/pmc/) and LOCKSS (www.lockss.org) repositories.

In addition, this published work and the nomenclatural acts it contains have been registered in ZooBank (www.zoobank.org), the proposed online registration system for the ICZN. The ZooBank LSIDs (Life Science Identifiers) can be resolved and the associated information viewed through any standard web browser by appending the LSID to the prefix “http://zoobank.org/”. The LSID for this publication is: urn:lsid:zoobank.org:pub:CC7DC93E-9BB6-4F1B-B96F-FD929E6FE1FD.

### DNA sequencing and phylogenetic methods

Whole genomic DNA was extracted from muscle or liver samples using a Qiagen DNeasy Blood & Tissue Kit (Qiagen, Inc. Valencia, CA, USA) as per manufacturer's instructions. A 700 bp fragment of the mitochondrial 12S ribosomal RNA gene and a 564 bp fragment of the mitochondrial 16S ribosomal RNA gene were amplified as in Austin *et al.* [39], but using an annealing temperature of 55°C for both genes and the primers L2519 and H3296 [40] or 16S-L and 16S-H [41] for 12S and 16S, respectively. PCR products were purified by incubation with Exonuclease I and Antarctic Phosphotase (New England Biolabs, Ipswich, MA, USA) as in Austin *et al.* [42], cycle sequenced in both directions using BigDye 3.1 (Applied Biosystems, Foster City, CA, USA) using previously published protocols [39], and sequenced on an ABI 3100 automated capillary sequencer (Applied Biosystems, Foster City, CA, USA).

Sequences were edited and complementary sequences were aligned using Sequencher ver. 4.7 (Gene Codes Corp., Ann Arbor, MI, USA). Genbank accession numbers for all sequences collected for this study are available in Table S1. These sequences were combined with previously published sequences (Table S1), resulting in a final dataset of 184 samples, including representatives of 9 of the 11 subfamilies of Microhylidae and all 22 genera in the subfamily Asterophryninae (which includes all New Guinean microhylids), as well as representatives of 4 non-microhylid, outgroup families (Arthroleptidae, Hyperoliidae, Hemisotidae, Brevicipitidae). The final dataset also includes a total of 70 genetypes, including 3 hologenetypes, 43 paragenetypes, and 24 topogenetypes (see Chakrabarty for details of nomenclature for sequences from type specimens [43]). Sequences were aligned in ClustalX2 [44] under default parameters (Gap opening penalty = 15, Gap extension penalty = 6.66). Some hyper-variable regions contained numerous indels, and thus could not be aligned with confidence, and were removed from subsequent analyses. The final concatenated and aligned dataset consisted of 925 bp (516 bp of 12S and 409 bp of 16S). The corrected Akaike Information Criterion was implemented in jModelTest ver. 0.1.1 [45] to select the best fit model of nucleotide substitution (GTR+I+G).

Phylogenetic relationships among sampled taxa were estimated using maximum likelihood (ML) and Bayesian (BI) analyses. Maximum likelihood analyses were conducted in Garli ver. 1.0 [46] with 50 search replicates; ML support was estimated with 1000 bootstrap pseudoreplicates, each with two search replicates. Bayesian analyses were implemented in Mr.Bayes ver. 3.1.2 [47], [48] with the nucleotide state frequencies and substitution rate priors set as flat Dirichlet distributions, and the proportion of invariable sites set as a uniform (0.0–1.0) prior distribution. Analyses consisted of two independent runs, each with four chains with default heating and sampling every 1,000 generations for 20,000,000 generations. Convergence was assessed by examining the potential scale reduction factors (all of which were close to 1 at run completion), by examining posterior probability, log likelihood, and all model parameters for stationary and by the effective sample sizes (ESSs) in Tracer ver. 1.5 [49] (all parameters were stationary with ESSs substantially greater than 200 at run completion), and by comparing the posterior probabilities of all splits between runs in Are We There Yet [50] (which were linear, supporting convergence of runs).

### Ancestral State Reconstructions

To examine the evolution of body size in asterophryine frogs, we used weighted squared-change parsimony [51], which is computationally equivalent to maximum likelihood based ancestral state reconstructions [52], [53], as implemented in Mesquite v.2.72 [54]. The maximum likelihood phylogeny of asterophryine frogs (Fig. S1) was trimmed to a single representative per generic-level clade for use in ancestral state reconstructions. We tested several different measures of body size for each clade, including mean size, maximum size of the smallest species, and maximum size of the largest species. Results did not differ substantially among analyses (data not shown), thus the results of ancestral state reconstructions with mean size for each clade are shown (Fig. 3). Mean size for each clade used in the analysis are provided in Table 6.

**Table 6:** Average sizes of asterophryine genera.

Genus/Clade	Representative Taxon	Mean SVL (mm)	References
*Albericus*	*Albericus laurini*	18.97	[68]–[71]
*Aphantophryne*	*Aphantophryne pansa*	22.27	[20], [68]
*Asterophrys*	*Asterophrys turpicola*	56.00	[68]
*Austrochaperina*	*Austrochaperina derongo*	29.60	[68], [72], [73]
*Austrochaperina*	*Austrochaperina guttata*	29.60	[68], [72], [73]
*Austrochaperina palmipes*	*Austrochaperina palmipes*	38.00	[68], [72]
*Barygenys*	*Barygenys exsul*	35.09	[68], [74], [75]
*Callulops*	*Callulops robustus*	56.42	[68], [76]
*Choerophryne*	*Choerophryne rostellifer*	16.89	[23], [24], [68], [77]
*Cophixalus*	*Cophixalus balbus*	25.00	[68], [70], [78]–[84]
*Cophixalus sphagnicola*	*Cophixalus sphagnicola*	17.85	[20], [68], [85]
*Cophixalus ateles* group	*Cophixalus tridactylus*	16.57	[68], [78], [82], [86]
*Copiula major*	*Copiula major*	43.00	[68]
*Copiula*	*Copiula obsti*	27.17	[68]
*Genyophryne*	*Genyophryne thomsoni*	40.00	[68]
*Hylophorbus*	*Hylophorbus wondiwoi*	31.26	[68], [76], [87]
*Liophryne dentata*	*Liophryne dentata*	38.00	[68], [72]
*Liophryne rhododactyla*	*Liophryne rhododactyla*	52.30	[68], [72]
*Liophryne schlaginhaufeni*	*Liophryne schlaginhaufeni*	38.00	[68], [72]
*Mantophryne*	*Mantophryne lateralis*	55.48	[68], [76], [88]
*Metamagnusia*	*Metamagnusia marani*	52.15	[68], [89]
*Oreophryne*	*Oreophryne atrigularis*	25.88	[22], [68], [70], [90]–[95]
*Oxydactyla*	*Oxydactyla crassa*	27.74	[72]
*Paedophryne amauensis*	*Paedophryne amauensis*	8.00	This Study
*Paedophryne oyatabu*	*Paedophryne oyatabu*	11.30	[13]
*Paedophryne swiftorum*	*Paedophryne swiftorum*	8.90	This Study
*Pherohapsis*	*Pherohapsis menziesi*	31.00	[68], [88]
*Pseudocallulops*	*Pseudocallulops pullifer*	35.75	[68], [88], [89]
*Sphenophryne*	*Sphenophryne cornuta*	37.40	[68], [88]
*Xenorhina* (+*Xenobatrachus*)	*Xenorhina bouwensi*	43.39	[68]

Average sizes (snout-to-vent length, SVL) of asterophryine genera used in reconstructions of ancestral body sizes.

### Morphology

Specimens of *Paedophryne amauensis* and *P. swiftorum*, with the exception of one individual (BPBM 31885, *P. swiftorum*, unsexed juvenile), were identified as mature males by the observation of calling behavior. Measurements, terminology, and abbreviations follow Kraus [13]: body length (SVL), tibia length (TL), horizontal eye diameter (EY), distance from anterior of eye to naris (EN), internarial distance between external nares (IN), distance from anterior of eye to tip of snout (SN), head width at center of tympana (HW), head length from posterior of tympana to tip of snout (HL), width of third finger disc (3F), and width of fourth toe disc (4T). All measurements were made to the nearest 0.05 mm using dial calipers or an optical micrometer.

## Supporting Information

Figure S1 **Maximum likelihood phylogeny of asterophryine frogs.** **A.** Full phylogeny (not trimmed to single exemplar per clade) of asterophryine frogs based on maximum likelihood analysis of 925 bp of 12S and 16S rDNA sequences. Numbers on branches indicate branch support assessed by 1000 bootstrap pseudoreplicates, followed by Bayesian posterior probability. Asterisks (*) indicate bootstrap support of 100 or posterior probability of 1.0. **B.** Full phylogeny of asterophryine frogs continued from Figure S1A.
(TIF)

Table S1 **Samples included in molecular phylogenetic analyses.** Specimens and Genbank accession numbers for samples used in phylogenetic analyses. Bolded lettering indicates sequences collected for this study.
(PDF)

## References

[1] Branch TA, Abubaker EMN, Mkango S, Butterworth DS (2007). Separating southern blue whale subspecies based on length frequencies of sexually mature females.. Marine Mammal Science.

[2] Kottelat M, Britz R, Hui TH, Witte K-E (2006). *Paedocypris*, a new genus of Southeast Asian cyprinid fish with a remarkable sexual dimorphism, comprises the world's smallest vertebrate.. Proceedings of the Royal Society B.

[3] Henderson DM (2004). Tipsy punters: sauropod dinosaur pneumaticity, buoyancy and aquatic habits.. Proceedings of the Royal Society of London B.

[4] Sander PM, Christian A, Clauss M, Fechner R, Gee CT (2011). Biology of sauropod dinosaurs: the evolution of gigantism.. Biological Reviews.

[5] Blanckenhorn WU (2000). The evolution of body size: what keeps organisms small?. The Quarterly Review of Biology.

[6] Hanken J, Wake DB (1993). Miniaturization of body size: organismal and evolutionary Significance.. Annual Review of Ecology and Systematics.

[7] Estrada AR, Hedges SB (1996). At the lower size limit in tetrapods: a new diminutive frog from Cuba (Leptodactylidae: *Eleutherodactylus*).. Copeia.

[8] Yeh J (2002). The effect of miniaturized body size on skeletal morphology in frogs.. Evolution.

[9] Trueb L, Alberch P, Duncker HR, Fleischer G (1985). Miniaturization and the anuran skull: a case study of heterochrony.. Functional morphology of the vertebrates.

[10] Duellman WE, Trueb L (1986). Biology of Amphibians.

[11] Kerney R, Meegaskumbura M, Manamendra-Arachchi K, Hanken J (2007). Cranial ontogeny in *Philautus silus* (Anura: Ranidae: Rhacophorinae) reveals few similarities with other direct-developing anurans.. Journal of Morphology.

[12] Hanken J, Klymkowsky MW, Summers CH, Seufert DW, Ingebrigsten N (1992). Cranial ontogeny in the direct-developing frog, *Eleutherodactylus coqui* (Anura: Leptodactyliae), analyzed using whole-mount immunohistochemistry.. Journal of Morphology.

[13] Kraus F (2010). New genus of diminutive microhylid frogs from Papua New Guinea.. ZooKeys.

[14] Hedges SB, Thomas R (2001). At the lower size limit in amniote vertebrates: a new diminutive lizard from the West Indies.. Caribbean Journal of Science.

[15] Hanken J, Wake DB (1994). Five new species of minute salamanders, genus *Thorius* (Caudata: Plethodontidae), from Northern Oaxaca, Mexico.. Copeia.

[16] Lehr E, Coloma LA (2008). A minute new Ecuadorian Andean frog (Anura: Strambomantidae, *Pristimantis*).. Herpetologica.

[17] Lehr E, Catenazzi A (2009). A new species of minute *Noblella* (Anura: Strambomantidae) from southern Peru: the smallest frog of the Andes.. Copeia.

[18] Nelson CE (1975). Another new miniature 4-toed South American microhylid frog (genus: *Syncope*).. Journal of Herpetology.

[19] Duellman WE, Mendelson JR (1995). Amphibians and reptiles from northern Departamento Loreto, Peru: taxonomy and biogeography.. University of Kansas Science Bulletin.

[20] Zweifel RG, Parker F (1989). New species of microhylid frogs from the Owen Stanley Mountains of Papua New Guinea and resurrection of the genus *Aphantophryne*.. American Museum Novitates.

[21] Matsui M (2011). Taxonomic revision of one of the Old World's smallest frogs, with description of a new Bornean *Microhyla* (Amphibia, Microhylidae).. Zootaxa.

[22] Richards SJ, Iskandar D (2000). A new minute *Oreophryne* (Anura: Microhylidae) from the mountains of Irian Jaya, Indonesia.. Raffles Bulletin of Zoology.

[23] Richards SJ, Burton TC (2003). A new species of *Choerophryne* (Anura: Microhylidae) from Southern Highlands Province, Papua New Guinea.. Transactions of the Royal Society of South Australia.

[24] Richards SJ, Dahl CS, Hiaso J (2007). Another new species of *Choerophryne* (Anura: Microhylidae) from Southern Highlands Province, Papua New Guinea.. Transactions of the Royal Society of South Australia.

[25] Young JE, Christian KA, Donnellan S, Tracy CR, Parry D (2005). Comparative analysis of cutaneous evaporative water loss in frogs demonstrates correlation with ecological habits.. Physiological and Biochemical Zoology.

[26] Young JE, Tracy CR, Christian KA, McArthur LJ (2006). Rates of cutaneous evaporative water loss of native Fijian frogs.. Copeia.

[27] Tracy CR, Christian KA, Betts G, Tracy CR (2008). Body temperature and resistance to evaporative water loss in tropical Australian frogs.. Comparative Biochemistry and Physiology - Part A: Molecular and Integrative Physiology.

[28] MacLean WP (1985). Water-loss rates of *Sphaerodactylus parthenopion* (Reptilia: Gekkonidae), the smallest amniote vertebrate.. Comparative Biochemistry and Physiology.

[29] Wells KD (2007). The Ecology & Behavior of Amphibians.

[30] Wiens JJ, Graham CH, Moen DS, Smith SA, Reeder TW (2006). Evolutionary and ecological causes of the latitudinal diversity gradient in hylid frogs: treefrog trees unearth the roots of high tropical diversity.. American Naturalist.

[31] Wiens JJ, Graham CH (2005). Niche conservatism: integrating evolution, ecology, and conservation biology.. Annual Review of Ecology, Evolution, and Systematics.

[32] Wiens JJ, Donoghue MJ (2004). Historical biogeography, ecology and species richness.. Trends in Ecology and Evolution.

[33] Toledo LF (2005). Predation of juvenile and adult anurans by invertebrates: current knowledge and prospects.. Herpetological Review.

[34] Pombal JP (2007). Predation notes in an anuran amphibians assemblage from southeastern Brazil.. Revista Brasileira de Zoologia.

[35] Toledo LF, Ribeiro RS, Haddad CFB (2007). Anurans as prey: an exploratory analysis and size relationships between predators and their prey.. Journal of Zoology.

[36] Davies H, Perembo R, Winn R, KenGemar P, Hancock G (1997). Terranes of the New Guinea Orogen.. Proceedings of the Geology Exploration and Mining Conference Madang.

[37] Davies H, Winn R, KenGemar P, P B (1996). Evolution of the Papuan Basin - a view from the orogen.. Petroleum exploration, development and production in Papua New Guinea.

[38] Hall R (2002). Cenozoic geological and plate tectonic evolution of SE Asia and the SW Pacific: computer-based reconstructions, model and animations.. Journal of Asian Earth Sciences.

[39] Austin CC, Spataro M, Peterson S, Jordan J, McVay JD (2010). Conservation genetics of Boelen's python (*Morelia boeleni*) from New Guinea: reduced genetic diversity and divergence of captive and wild animals. Conservation Genetics.

[40] Richards CM, Moore WS (1996). A phylogeny for the African treefrog family Hyperoliidae based on mitochondrial rDNA.. Molecular Phylogenetics and Evolution.

[41] Köhler F, Günther R (2008). The radiation of microhylid frogs (Amphibia: Anura) on New Guinea: a mitochondrial phylogeny reveals parallel evolution of morphological and life history traits and disproves the current morphology-based classification.. Molecular Phylogenetics and Evolution.

[42] Austin CC, Rittmeyer EN, Richards SJ, Zug GR (2010). Phylogeny, historical biogeography and body size evolution in Pacific Island Crocodile skinks *Tribolonotus* (Squamata; Scincidae).. Molecular Phylogenetics and Evolution.

[43] Chakrabarty P (2010). Genetypes: a concept to help integrate molecular phylogenetics and taxonomy.. Zootaxa.

[44] Larkin MA, Blackshields G, Brown NP, Chenna R, McGettigan PA (2007). Clustal W and Clustal X version 2.0.. Bioinformatics.

[45] Posada D (2008). jModelTest: phylogenetic model averaging.. Molecular Biology and Evolution.

[46] Zwickl DJ (2006). Genetic algorithm approaches for the phylogenetic analysis of large biological sequence datasets under the maximum likelihood criterion: The University of Texas at Austin..

[47] Huelsenbeck JP, Ronquist F (2001). MRBAYES: Bayesian inference of phylogeny.. Bioinformatics.

[48] Ronquist F, Huelsenbeck JP (2003). MRBAYES 3: Bayesian phylogenetic inference under mixed models.. Bioinformatics.

[49] Rambaut A, Drummond AJ (2007). http://beast.bio.ed.ac.uk/Tracer.

[50] Nylander JAA, Wilgenbusch JC, Warren DL, Swofford DL (2008). AWTY (are we there yet?): a system for graphical exploration of MCMC convergence in Bayesian phylogenetics.. Bioinformatics.

[51] Maddison WP (1991). Squared-change parsimony reconstructions of ancestral states for continuous-valued characters on a phylogenetic tree.. Systematic Zoology.

[52] Schluter D, Price T, Mooers AO, Ludwig D (1997). Likelihood of ancestral states in adaptive radiation.. Evolution.

[53] Martins EP (1999). Estimation of ancestral states of continuous characters: a computer simulation study.. Systematic Biology.

[54] Maddison WP, Maddison DR (2009). Mesquite: a modular system for evolutionary analysis.. http://mesquiteproject.org.

[55] Almeida-Santos M, Siqueira CC, Van Sluys M, Rocha CFD (2011). Ecology of the Brazilian Flea Frog *Brachycephalus didactylus* (Terrarana: Brachycephalidae).. Journal of Herpetology.

[56] Krüger P, Richter S (1995). *Syncope antenori* - a bromeliad breeding frog with free-swimming, nonfeeding tadpoles (Anura, Microhylidae).. Copeia.

[57] Giaretta AA, Sawaya RJ (1998). Second species of *Psyllophryne* (Anura: Brachycephalidae).. Copeia.

[58] Van Der Meijden A, Boistel R, Gerlach J, Ohler A, Vences M (2007). Molecular phylogenetic evidence for paraphyly of the genus *Sooglossus*, with the description of a new genus of Seychellean frogs.. Biological Journal of the Linnean Society.

[59] Glaw F, Vences M (1992). A Field Guide to the Amphibians and Reptiles of Madagascar.

[60] Walker CF (1973). A new genus and species of microhylid frog from Ecuador.. Occasional Papers of the Museum of Natural History, University of Kansas.

[61] Ribeiro LF, Alves ACR, Haddad CFB, Dos Reis SF (2005). Two new species of *Brachycephalus* Günther, 1858 from the state of Paraná, Southern Brazil (Amphibia, Anura, Brachycephalidae).. Boletim Do Museu Nacional.

[62] Lynch JD (1976). Two new species of frogs of the genus *Euparkerella* (Amphibia: Leptodactylidae) from Ecuador and Peru.. Herpetologica.

[63] Heyer WR, Rand AS, da Cruz CAG, Peixoto OL, Nelson CE (1990). Frogs of Bracéia.. Arquivos do Zoologica, Museu de Zoologia da Universidade de São Paulo.

[64] Inger RF, Frogner KJ (1979). New species of narrow-mouth frogs (genus *Microhyla*) from Borneo.. Sarawak Museum Journal.

[65] Estrada AR, Hedges SB (1996). A new frog of the genus *Eleutherodactylus* from eastern Cuba (Anura: Leptodactylidae).. Herpetologica.

[66] Hedges SB (1988). A new diminutive frog from Hispaniola (Leptodactylidae: *Eleutherodactylus*).. Copeia.

[67] Alves ACR, Ribeiro LF, Haddad CFB, Dos Reis SF (2006). Two new species of *Brachycephalus* (Anura: Brachycephalidae) from the Atlantic forest in Paraná State, Southern Brazil.. Herpetologica.

[68] Menzies JI (2006). Frogs of New Guinea and the Solomon Islands: Pensoft Publishers..

[69] Günther R (2000). *Albericus laurini* species nova, the first record of the genus *Albericus* (Anura, Microhylidae) from the west of New Guinea.. Mitteilungen aus dem Museum fur Naturkunde in Berlin Zoologische Reihe.

[70] Kraus F, Allison A (2009). New microhylid frogs from the Muller Range, Papua New Guinea.. ZooKeys.

[71] Kraus F, Allison A (2005). New species of *Albericus* (Anura: Microhylidae) from eastern New Guinea.. Copeia.

[72] Zweifel RG (2000). Partition of the Australopapuan microhylid genus *Sphenophryne* with descriptions of new species.. Bulletin of the American Museum of Natural History.

[73] Günther R (2009). A new and minute species of *Austrochaperina* (Amphibia: Anura: Microhylidae) from western New Guinea.. Vertebrate Zoology.

[74] Zweifel RG (1963). Results of the Archbold Expeditions. No. 84. New microhylid frogs (*Baragenys* and *Cophixalus*) from the Louisiade Archipelago, New Guinea.. American Museum Novitates.

[75] Zweifel RG (1980). Description and relationships of a new species of microhylid frog (genus *Barygenys*) from Papua New Guinea.. Pacific Science.

[76] Kraus F, Allison A (2009). New species of frogs from Papua New Guinea.. Bishop Museum Occasional Papers.

[77] Günther R (2008). Descriptions of four new species of *Choerophryne* (Anura, Microhylidae) from Papua Province, Indonesian New Guinea.. Acta Zoologica Sinica.

[78] Kraus F, Allison A (2009). New species of *Cophixalus* (Anura: Microhylidae) from Papua New Guinea.. Zootaxa.

[79] Richards SJ, Oliver PM (2007). A new species of *Cophixalus* (Anura: Microhylidae) from Misima Island, Papua New Guinea.. Pacific Science.

[80] Zweifel RG (1979). A new cryptic species of microhylid frog (genus *Cophixalus*) from Papua New Guinea, with notes on related forms.. American Museum Novitates.

[81] Menzies JI (1976). Handbook of common New Guinea frogs.

[82] Kraus F, Allison A (2006). Three new species of *Cophixalus* (Anura: Microhylidae) from southeastern New Guinea.. Herpetologica.

[83] Günther R (2010). Another new *Cophixalus* species (Amphibia: Anura: Microhylidae) from western New Guinea.. Bonn Zoological Bulletin.

[84] Richards SJ, Oliver PM (2010). A new scansorial species of *Cophixalus* (Anura: Microhylidae) from the Kikori River basin, Papua New Guinea.. Journal of Herpetology.

[85] Zweifel RG, Allison A (1982). A new montane microhylid frog from Papua New Guinea with comments on the status of the genus *Aphantophryne*.. American Museum Novitates.

[86] Günther R (2006). Two new tiny *Cophixalus* species with reduced thumbs from the west of New Guinea (Anura: Microhylidae).. Herpetozoa.

[87] Richards SJ, Oliver PM (2007). A new species of *Hylophorbus* (Anura, Microhylidae) from the Huon Peninsula, Papua New Guinea.. Mitteilungen aus dem Museum fur Naturkunde in Berlin Zoologische Reihe.

[88] Zweifel RG (1972). Results of the Archbold Expeditions. No. 97. A revision of the frogs of the subfamily Asterophryinae, family Microhylidae.. Bulletin of the American Museum of Natural History.

[89] Günther R (2009). *Metamagnusia* and *Pseudocallulops*, two new genera of microhylid frogs from New Guinea (Amphibia, Anura, Microhylidae).. Zoosystematics and Evolution.

[90] Zweifel RG, Menzies JI, Price D (2003). Systematics of microhylid frogs, genus *Oreophryne*, from the north coast region of New Guinea.. American Museum Novitates.

[91] Günther R, Richards SJ, Iskandar D (2001). Two new species of the genus *Oreophryne* from Irian Jaya, Indonesia.. Spixiana.

[92] Zweifel RG, Cogger HG, Richards SJ (2005). Systematics of microhylid frogs, genus *Oreophryne*, living at high elevations in New Guinea.. American Museum Novitates.

[93] Zweifel RG (2003). A new species of microhylid frog, genus *Oreophryne*, from Papua New Guinea.. American Museum Novitates.

[94] Kraus F, Allison A (2009). A remarkable ontogenetic change in color pattern in a new species of *Oreophryne* (Anura: Microhylidae) from Papua New Guinea.. Copeia.

[95] Günther R, Richards SJ, Tjaturadi B, Iskandar D (2009). A new species of the microhylid frog genus *Oreophryne* from the Mamberamo basin of northern Papua Province, Indonesian New Guinea.. Vertebrate Zoology.

