# Supplementary figures and images for: Ecological Guild Evolution and the Discovery of the World's Smallest Vertebrate
Source: PLoS One. 2012 Jan 11;7(1):e29797. doi: 10.1371/journal.pone.0029797 (PMC3256195; doi:10.1371/journal.pone.0029797)

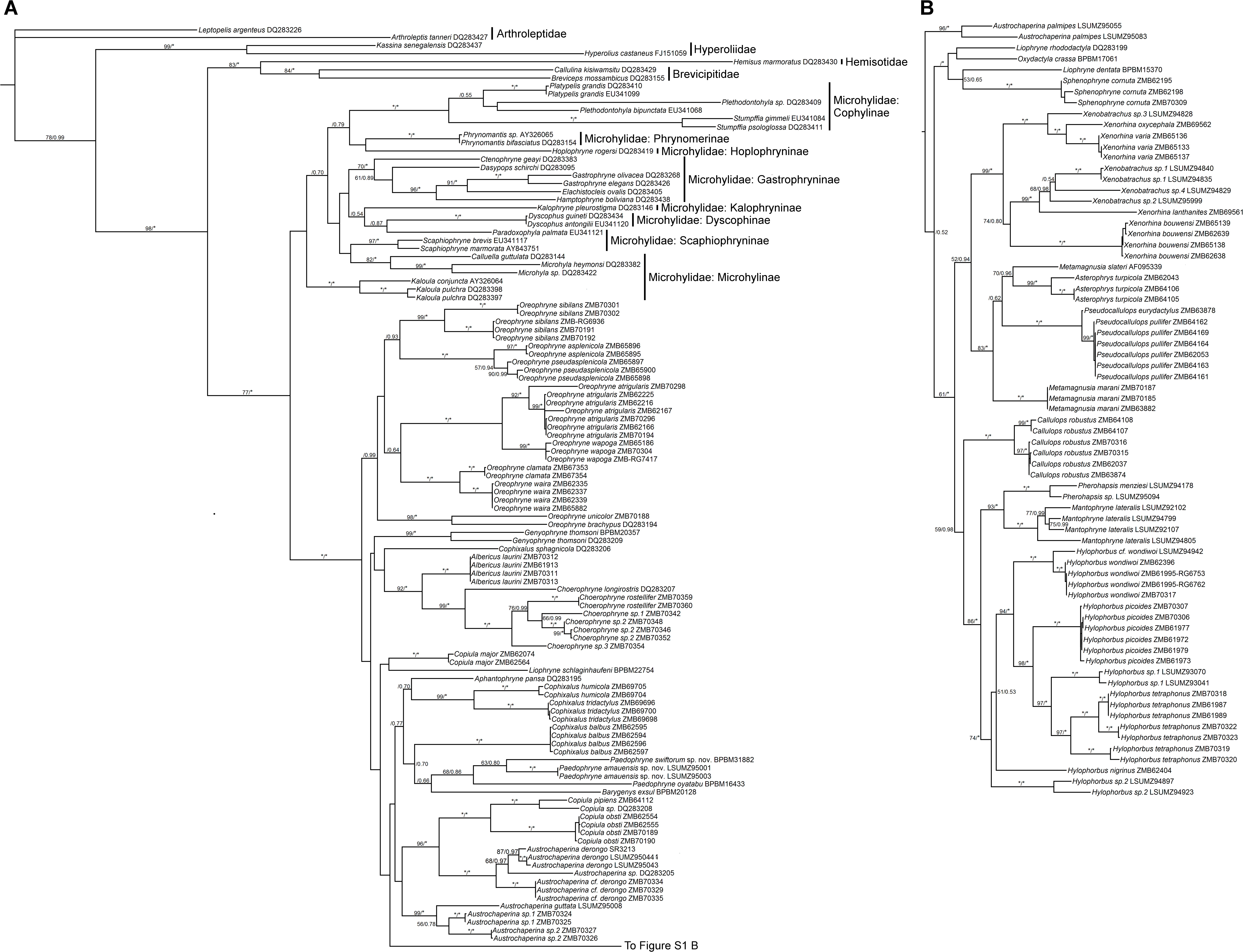

Supplement: Figure S1 — Maximum likelihood phylogeny of asterophryine frogs. A. Full phylogeny (not trimmed to single exemplar per clade) of asterophryine frogs based on maximum likelihood analysis of 925 bp of 12S and 16S rDNA sequences. Numbers on branches indicate branch support assessed by 1000 bootstrap pseudoreplicates, followed by Bayesian posterior probability. Asterisks (*) indicate bootstrap support of 100 or posterior probability of 1.0. B. Full phylogeny of asterophryine frogs continued from Figure S1A. (TIF) [file pone.0029797.s001.tif]
