# Supplementary material for: Ecological Guild Evolution and the Discovery of the World's Smallest Vertebrate
Source: PLoS One. 2012 Jan 11;7(1):e29797. doi: 10.1371/journal.pone.0029797 (PMC3256195; doi:10.1371/journal.pone.0029797)
Supplement: Table S1 — Samples included in molecular phylogenetic analyses. Specimens and Genbank accession numbers for samples used in phylogenetic analyses. Bolded lettering indicates sequences collected for this study. (PDF) [file pone.0029797.s002.pdf]

Table S1. Samples included in molecular phylogenetic analyses. Specimens and Genbank accession numbers for samples used in phylogenetic analyses. Bolded lettering indicates sequences collected for this study.

						**GenBank Accession No.**	
**Family**	**Subfamily**	**Genus**	**Species**	**Locality**	**Voucher No.**	**12S**	**16S**
Arthroleptidae	Arthroleptinae	*Arthroleptis*	*tanneri*	Tanzania: West Usumbaras Mts	RdS 929	DQ283427	DQ283427
Arthroleptidae	Leptopelinae	*Leptopelis*	*argenteus*	Kenya: Kilifi District	CAS 169938	DQ283226	DQ283226
Brevicipitidae	—	*Breviceps*	*mossambicus*	Tanzania: Morogoro	RdS 903	DQ283155	DQ283155
Brevicipitidae	—	*Callulina*	*kisiwamsitu*	Tanzania: West Usumbara Mts.	RdS936	DQ283429	DQ283429
Hemisotidae	—	*Hemisus*	*marmoratus*	Tanzania: Arusha: Masai Camp	RdS 916	DQ283430	DQ283430
Hyperoliidae	—	*Hyperolius*	*castaneus*	Uganda: Kabale Dist.	CAS 202372	FJ151059	FJ151059
Hyperoliidae	—	*Kassina*	*senegalensis*	Tanzania: Iringa	RdS 803	DQ283437	DQ283437
Microhylidae	Asterophryinae	*Albericus*	*laurini*	WNG: Wondiwoi Mts.	ZMB 61913	EU100220	EU100104
Microhylidae	Asterophryinae	*Albericus*	*laurini*	WNG: Wondiwoi Mts.	ZMB 70311	EU100221	EU100105
Microhylidae	Asterophryinae	*Albericus*	*laurini*	WNG: Wondiwoi Mts.	ZMB 70312	EU100222	EU100106
Microhylidae	Asterophryinae	*Albericus*	*laurini*	WNG: Wondiwoi Mts.	ZMB 70313	EU100223	EU100107
Microhylidae	Asterophryinae	*Aphantophryne*	*pansa*	PNG: Morobe: Bulolo	ABTC 49605	DQ283195	DQ283195
Microhylidae	Asterophryinae	*Asterophrys*	*turpicola*	WNG: Wondiwoi Mts.	ZMB 62043	EU100224	EU100108
Microhylidae	Asterophryinae	*Asterophrys*	*turpicola*	WNG: Yapen Isl.	ZMB 64105	EU100225	EU100109
Microhylidae	Asterophryinae	*Asterophrys*	*turpicola*	WNG: Yapen Isl.	ZMB 64106	EU100226	EU100110
Microhylidae	Asterophryinae	*Austrochaperina*	*derongo*	WNG: Wapoga camp	SR3213	EU100229	EU100113
**Microhylidae**	**Asterophryinae**	***Austrochaperina***	***derongo***	**PNG: Gulf: Sobo**	**LSUMZ 95043**	**JN049006**	**JN048981**
**Microhylidae**	**Asterophryinae**	***Austrochaperina***	***derongo***	**PNG: Gulf: Sobo**	**LSUMZ 95044**	**JN049008**	**JN048983**
Microhylidae	Asterophryinae	*Austrochaperina*	*cf. derongo*	WNG: Yapen Isl.	ZMB 70334	EU100230	EU100114
Microhylidae	Asterophryinae	*Austrochaperina*	*cf. derongo*	WNG: Yapen Isl.	ZMB 70335	EU100231	EU100115
Microhylidae	Asterophryinae	*Austrochaperina*	*cf. derongo*	WNG: Yapen Isl.	ZMB 70329	EU100232	EU100116
**Microhylidae**	**Asterophryinae**	***Austrochaperina***	***guttata***	**PNG: Gulf: Sobo**	**LSUMZ 95008**	**JN049004**	**JN048979**
**Microhylidae**	**Asterophryinae**	***Austrochaperina***	***palmipes***	**PNG: Gulf: Sobo**	**LSUMZ 95083**	**JN049007**	**JN048982**
**Microhylidae**	**Asterophryinae**	***Austrochaperina***	***palmipes***	**PNG: Milne Bay: Alotau**	**LSUMZ 95055**	**JN049017**	**JN048992**
Microhylidae	Asterophryinae	*Austrochaperina*	*sp.*	PNG: S. Highlands: Namosado	AMS R122221	DQ283205	DQ283205
Microhylidae	Asterophryinae	*Austrochaperina*	*sp. 1*	WNG: Wondiwoi Mts.	ZMB 70324	EU100227	EU100111
Microhylidae	Asterophryinae	*Austrochaperina*	*sp. 1*	WNG: Wondiwoi Mts.	ZMB 70325	EU100228	EU100112
Microhylidae	Asterophryinae	*Austrochaperina*	*sp. 2*	WNG: Wondiwoi Mts.	ZMB 70326	EU100233	EU100117
Microhylidae	Asterophryinae	*Austrochaperina*	*sp. 2*	WNG: Wondiwoi Mts.	ZMB 70327	EU100234	EU100118
Microhylidae	Asterophryinae	*Barygenys*	*exsul*	PNG: Milne Bay: Rossel Isl.	BPBM 20128	EU100235	EU100119
Microhylidae	Asterophryinae	*Callulops*	*robustus*	WNG: Wondiwoi Mts.	ZMB 62037	EU100247	EU100131
Microhylidae	Asterophryinae	*Callulops*	*robustus*	WNG: Wondiwoi Mts.	ZMB 70315	EU100250	EU100134
Microhylidae	Asterophryinae	*Callulops*	*robustus*	WNG: Wondiwoi Mts.	ZMB 70316	EU100251	EU100135
Microhylidae	Asterophryinae	*Callulops*	*robustus*	WNG: Wondiwoi Mts.	ZMB 63874	EU100246	EU100130
Microhylidae	Asterophryinae	*Callulops*	*robustus*	WNG: Biak Isl.	ZMB 64107	EU100248	EU100132
Microhylidae	Asterophryinae	*Callulops*	*robustus*	WNG: Biak Isl.	ZMB 64108	EU100249	EU100133
Microhylidae	Asterophryinae	*Choerophryne*	*rostellifer*	WNG: Wondiwoi Mts.	ZMB 70359	EU100253	EU100137
Microhylidae	Asterophryinae	*Choerophryne*	*rostellifer*	WNG: Wondiwoi Mts.	ZMB 70360	EU100254	EU100138
Microhylidae	Asterophryinae	*Choerophryne*	*longirostris*	PNG: Sandaun: Mt. Menawa	ABTC 47720	DQ283207	DQ283207

Microhylidae	Asterophryinae	*Choerophryne*	*sp. 1*	WNG: Yapen Isl.	ZMB 70342	EU100252	EU100136
Microhylidae	Asterophryinae	*Choerophryne*	*sp. 2*	WNG: Yapen Isl.	ZMB 70346	EU100255	EU100139
Microhylidae	Asterophryinae	*Choerophryne*	*sp. 2*	WNG: Yapen Isl.	ZMB 70348	EU100256	EU100140
Microhylidae	Asterophryinae	*Choerophryne*	*sp. 2*	WNG: Yapen Isl.	ZMB 70352	EU100257	EU100141
Microhylidae	Asterophryinae	*Choerophryne*	*sp. 3*	WNG: Yapen Isl.	ZMB 70354	EU100258	EU100142
Microhylidae	Asterophryinae	*Cophixalus*	*balbus*	WNG: Yapen Isl.	ZMB 62594	EU100259	EU100143
Microhylidae	Asterophryinae	*Cophixalus*	*balbus*	WNG: Yapen Isl.	ZMB 62595	EU100260	EU100144
Microhylidae	Asterophryinae	*Cophixalus*	*balbus*	WNG: Yapen Isl.	ZMB 62596	EU100261	EU100145
Microhylidae	Asterophryinae	*Cophixalus*	*balbus*	WNG: Yapen Isl.	ZMB 62597	EU100262	EU100146
Microhylidae	Asterophryinae	*Cophixalus*	*humicola*	WNG: Yapen Isl.	ZMB 69704	EU100263	EU100147
Microhylidae	Asterophryinae	*Cophixalus*	*humicola*	WNG: Yapen Isl.	ZMB 69705	EU100264	EU100148
Microhylidae	Asterophryinae	*Cophixalus*	*sphagnicola*	PNG: Morobe: Wau	ABTC 47881	DQ283206	DQ283206
Microhylidae	Asterophryinae	*Cophixalus*	*tridactylus*	WNG: Wondiwoi Mts.	ZMB 69696	EU100265	EU100149
Microhylidae	Asterophryinae	*Cophixalus*	*tridactylus*	WNG: Wondiwoi Mts.	ZMB 69698	EU100266	EU100150
Microhylidae	Asterophryinae	*Cophixalus*	*tridactylus*	WNG: Wondiwoi Mts.	ZMB 69700	EU100267	EU100151
Microhylidae	Asterophryinae	*Copiula*	*major*	WNG: Wondiwoi Mts.	ZMB 62074	EU100268	EU100152
Microhylidae	Asterophryinae	*Copiula*	*major*	WNG: Wondiwoi Mts.	ZMB 62564	EU100269	EU100153
Microhylidae	Asterophryinae	*Copiula*	*obsti*	WNG: Yapen Isl.	ZMB 62555	EU100270	EU100154
Microhylidae	Asterophryinae	*Copiula*	*obsti*	WNG: Yapen Isl.	ZMB 62554	EU100271	EU100155
Microhylidae	Asterophryinae	*Copiula*	*obsti*	WNG: Yapen Isl.	ZMB 70189	EU100272	EU100156
Microhylidae	Asterophryinae	*Copiula*	*obsti*	WNG: Yapen Isl.	ZMB 70190	EU100273	EU100157
Microhylidae	Asterophryinae	*Copiula*	*pipiens*	WNG: Yapen Isl.	ZMB 64112	EU100274	EU100158
Microhylidae	Asterophryinae	*Copiula*	*sp.*	PNG: Madang: Sinyarge	AMS R124417	DQ283208	DQ283208
Microhylidae	Asterophryinae	*Genyophryne*	*thomsoni*	PNG: Milne Bay: Sudest Isl.	BPBM 20357	EU100275	EU100159
Microhylidae	Asterophryinae	*Genyophryne*	*thomsoni*	PNG: Morobe: Bulolo	ABTC 49624	DQ283209	DQ283209
Microhylidae	Asterophryinae	*Hylophorbus*	*nigrinus*	WNG: Yapen Isl.	ZMB 62404	EU100276	EU100160
Microhylidae	Asterophryinae	*Hylophorbus*	*picoides*	WNG: Wondiwoi Mts.	ZMB 61972	EU100278	EU100162
Microhylidae	Asterophryinae	*Hylophorbus*	*picoides*	WNG: Wondiwoi Mts.	ZMB 61979	EU100279	EU100163
Microhylidae	Asterophryinae	*Hylophorbus*	*picoides*	WNG: Wondiwoi Mts.	ZMB 61977	EU100280	EU100164
Microhylidae	Asterophryinae	*Hylophorbus*	*picoides*	WNG: Wondiwoi Mts.	ZMB 70306	EU100281	EU100165
Microhylidae	Asterophryinae	*Hylophorbus*	*picoides*	WNG: Wondiwoi Mts.	ZMB 70307	EU100282	EU100166
Microhylidae	Asterophryinae	*Hylophorbus*	*picoides*	WNG: Wondiwoi Mts.	ZMB 61973	EU100277	EU100161
Microhylidae	Asterophryinae	*Hylophorbus*	*tetraphonus*	WNG: Wondiwoi Mts.	ZMB 61987	EU100283	EU100167
Microhylidae	Asterophryinae	*Hylophorbus*	*tetraphonus*	WNG: Wondiwoi Mts.	ZMB 61989	EU100284	EU100168
Microhylidae	Asterophryinae	*Hylophorbus*	*tetraphonus*	WNG: Wondiwoi Mts.	ZMB 70318	EU100287	EU100171
Microhylidae	Asterophryinae	*Hylophorbus*	*tetraphonus*	WNG: Yapen Isl.	ZMB 70319	EU100285	EU100169
Microhylidae	Asterophryinae	*Hylophorbus*	*tetraphonus*	WNG: Yapen Isl.	ZMB 70320	EU100286	EU100170
Microhylidae	Asterophryinae	*Hylophorbus*	*tetraphonus*	WNG: Nabire-Mapia road	ZMB 70322	EU100288	EU100172
Microhylidae	Asterophryinae	*Hylophorbus*	*tetraphonus*	WNG: Nabire-Mapia road	ZMB 70323	EU100289	EU100173
Microhylidae	Asterophryinae	*Hylophorbus*	*wondiwoi*	WNG: Wondiwoi Mts.	ZMB 61995-RG67[illegible]	EU100290	EU100174
Microhylidae	Asterophryinae	*Hylophorbus*	*wondiwoi*	WNG: Wondiwoi Mts.	ZMB 61995-RG67[illegible]	EU100291	EU100175
Microhylidae	Asterophryinae	*Hylophorbus*	*wondiwoi*	WNG: Wondiwoi Mts.	ZMB 62396	EU100292	EU100176
Microhylidae	Asterophryinae	*Hylophorbus*	*wondiwoi*	WNG: Wondiwoi Mts.	ZMB 70317	EU100293	EU100177

**Microhylidae**	**Asterophryinae**	***Hylophorbus***	***cf. wondiwoi***	**PNG: Gulf: Sobo**	**LSUMZ 94942**	**JN049026**	**JN049001**
**Microhylidae**	**Asterophryinae**	***Hylophorbus***	***sp. 1***	**PNG: Sandaun: Utai**	**LSUMZ 93041**	**JN049011**	**JN048986**
**Microhylidae**	**Asterophryinae**	***Hylophorbus***	***sp. 1***	**PNG: Sandaun: Utai**	**LSUMZ 93070**	**JN049015**	**JN048990**
**Microhylidae**	**Asterophryinae**	***Hylophorbus***	***sp. 2***	**PNG: Milne Bay: Alotau**	**LSUMZ 94897**	**JN049018**	**JN048993**
**Microhylidae**	**Asterophryinae**	***Hylophorbus***	***sp. 2***	**PNG: Central: Amau**	**LSUMZ 94923**	**JN049022**	**JN048997**
Microhylidae	Asterophryinae	*Liophryne*	*dentata*	PNG: Milne Bay: Cloudy Mts.	BPBM 15370	EU100294	EU100178
Microhylidae	Asterophryinae	*Liophryne*	*rhododactyla*	PNG: Morobe: Bulolo	ABTC 49566	DQ283199	DQ283199
Microhylidae	Asterophryinae	*Liophryne*	*schlaginhaufeni*	PNG: Sandaun: Toricelli Mts.	BPBM 22754	EU100295	EU100179
**Microhylidae**	**Asterophryinae**	***Mantophryne***	***lateralis***	**PNG: Sandaun: Utai**	**LSUMZ 92102**	**JN049014**	**JN048989**
**Microhylidae**	**Asterophryinae**	***Mantophryne***	***lateralis***	**PNG: Milne Bay: Halowia**	**LSUMZ 92107**	**JN049016**	**JN048991**
**Microhylidae**	**Asterophryinae**	***Mantophryne***	***lateralis***	**PNG: Central: Amau**	**LSUMZ 94799**	**JN049020**	**JN048995**
**Microhylidae**	**Asterophryinae**	***Mantophryne***	***lateralis***	**PNG: Gulf: Sobo**	**LSUMZ 94805**	**JN049025**	**JN049000**
Microhylidae	Asterophryinae	*Metamagnusia*	*marani*	WNG: Wondiwoi Mts.	ZMB 63882	EU100237	EU100121
Microhylidae	Asterophryinae	*Metamagnusia*	*marani*	WNG: Wondiwoi Mts.	ZMB 70187	EU100238	EU100122
Microhylidae	Asterophryinae	*Metamagnusia*	*marani*	WNG: Wondiwoi Mts.	ZMB 70185	EU100239	EU100123
Microhylidae	Asterophryinae	*Metamagnusia*	*slateri*	—	—	AF095339	—
Microhylidae	Asterophryinae	*Oreophryne*	*asplenicola*	WNG: Yapen Isl.	ZMB 65895	EU100296	EU100180
Microhylidae	Asterophryinae	*Oreophryne*	*asplenicola*	WNG: Yapen Isl.	ZMB 65896	EU100297	EU100181
Microhylidae	Asterophryinae	*Oreophryne*	*atrigularis*	WNG: Wondiwoi Mts.	ZMB 62216	EU100298	EU100182
Microhylidae	Asterophryinae	*Oreophryne*	*atrigularis*	WNG: Wondiwoi Mts.	ZMB 62225	EU100299	EU100183
Microhylidae	Asterophryinae	*Oreophryne*	*atrigularis*	WNG: Wondiwoi Mts.	ZMB 62166	EU100300	EU100184
Microhylidae	Asterophryinae	*Oreophryne*	*atrigularis*	WNG: Wondiwoi Mts.	ZMB 62167	EU100301	EU100185
Microhylidae	Asterophryinae	*Oreophryne*	*atrigularis*	WNG: Wondiwoi Mts.	ZMB 70296	EU100302	EU100186
Microhylidae	Asterophryinae	*Oreophryne*	*atrigularis*	WNG: Wondiwoi Mts.	ZMB 70194	EU100303	EU100187
Microhylidae	Asterophryinae	*Oreophryne*	*atrigularis*	WNG: Nabire-Mapia road	ZMB 70298	EU100304	EU100188
Microhylidae	Asterophryinae	*Oreophryne*	*brachypus*	PNG: W. New Britain: near Amelei	AMS R129618	DQ283194	DQ283194
Microhylidae	Asterophryinae	*Oreophryne*	*clamata*	WNG: Wondiwoi Mts.	ZMB 67353	EU100305	EU100189
Microhylidae	Asterophryinae	*Oreophryne*	*clamata*	WNG: Wondiwoi Mts.	ZMB 67354	EU100306	EU100190
Microhylidae	Asterophryinae	*Oreophryne*	*pseudasplenicola*	WNG: Yapen Isl.	ZMB 65897	EU100307	EU100191
Microhylidae	Asterophryinae	*Oreophryne*	*pseudasplenicola*	WNG: Yapen Isl.	ZMB 65898	EU100308	EU100192
Microhylidae	Asterophryinae	*Oreophryne*	*pseudasplenicola*	WNG: Yapen Isl.	ZMB 65900	EU100309	EU100193
Microhylidae	Asterophryinae	*Oreophryne*	*sibilans*	WNG: Wondiwoi Mts.	ZMB-RG6936	EU100310	EU100194
Microhylidae	Asterophryinae	*Oreophryne*	*sibilans*	WNG: Wondiwoi Mts.	ZMB 70191	EU100313	EU100197
Microhylidae	Asterophryinae	*Oreophryne*	*sibilans*	WNG: Wondiwoi Mts.	ZMB 70192	EU100314	EU100198
Microhylidae	Asterophryinae	*Oreophryne*	*sibilans*	WNG: Yapen Isl.	ZMB 70301	EU100311	EU100195
Microhylidae	Asterophryinae	*Oreophryne*	*sibilans*	WNG: Yapen Isl.	ZMB 70302	EU100312	EU100196
Microhylidae	Asterophryinae	*Oreophryne*	*unicolor*	WNG: Wondiwoi Mts.	ZMB 70188	EU100315	EU100199
Microhylidae	Asterophryinae	*Oreophryne*	*waira*	WNG: Yapen Isl.	ZMB 65882	EU100316	EU100200
Microhylidae	Asterophryinae	*Oreophryne*	*waira*	WNG: Yapen Isl.	ZMB 62337	EU100317	EU100201
Microhylidae	Asterophryinae	*Oreophryne*	*waira*	WNG: Yapen Isl.	ZMB 62339	EU100318	EU100202
Microhylidae	Asterophryinae	*Oreophryne*	*waira*	WNG: Yapen Isl.	ZMB 62335	EU100319	EU100203
Microhylidae	Asterophryinae	*Oreophryne*	*cf. wapoga*	WNG: Yapen Isl.	ZMB 65186	EU100320	EU100204
Microhylidae	Asterophryinae	*Oreophryne*	*cf. wapoga*	WNG: Yapen Isl.	ZMB-RG7417	EU100321	EU100205

Microhylidae	Asterophryinae	*Oreophryne*	*cf. wapoga*	WNG: Yapen Isl.	ZMB 70304	EU100322	EU100206
Microhylidae	Asterophryinae	*Oxydactyla*	*crassa*	PNG: Milne Bay: Mt. Simpson	BPBM 17061	EU100323	EU100207
**Microhylidae**	**Asterophryinae**	***Paedophryne***	***amauensis sp. nov.***	**PNG: Central: Amau**	**LSUMZ 95001**	**JN049021**	**JN048996**
**Microhylidae**	**Asterophryinae**	***Paedophryne***	***amauensis sp. nov.***	**PNG: Central: Amau**	**LSUMZ 95003**	**JN049023**	**JN048998**
**Microhylidae**	**Asterophryinae**	***Paedophryne***	***oyatabu***	**PNG: Milne Bay: Fergusson Isl.**	**BPBM 16433**	**—**	**JN049002**
**Microhylidae**	**Asterophryinae**	***Paedophryne***	***swiftorum sp. nov.***	**PNG: Morobe: Kamiali**	**BPBM 31882**	**JN049003**	**JN048978**
**Microhylidae**	**Asterophryinae**	***Pherohapsis***	***menziesi***	**PNG: NCD: Brown River**	**LSUMZ 94178**	**JN049010**	**JN048985**
**Microhylidae**	**Asterophryinae**	***Pherohapsis***	***sp.***	**PNG: Central: Amau**	**LSUMZ 95094**	**JN049019**	**JN048994**
Microhylidae	Asterophryinae	*Pseudocallulops*	*eurydactylus*	WNG: Fakfak Mts.	ZMB 63878	EU100236	EU100120
Microhylidae	Asterophryinae	*Pseudocallulops*	*pullifer*	WNG: Wondiwoi Mts.	ZMB 62053	EU100240	EU100124
Microhylidae	Asterophryinae	*Pseudocallulops*	*pullifer*	WNG: Wondiwoi Mts.	ZMB 64161	EU100241	EU100125
Microhylidae	Asterophryinae	*Pseudocallulops*	*pullifer*	WNG: Wondiwoi Mts.	ZMB 64162	EU100242	EU100126
Microhylidae	Asterophryinae	*Pseudocallulops*	*pullifer*	WNG: Wondiwoi Mts.	ZMB 64163	EU100243	EU100127
Microhylidae	Asterophryinae	*Pseudocallulops*	*pullifer*	WNG: Wondiwoi Mts.	ZMB 64164	EU100244	EU100128
Microhylidae	Asterophryinae	*Pseudocallulops*	*pullifer*	WNG: Wondiwoi Mts.	ZMB 64169	EU100245	EU100129
Microhylidae	Asterophryinae	*Sphenophryne*	*cornuta*	WNG: Wondiwoi Mts.	ZMB 62195	EU100324	EU100208
Microhylidae	Asterophryinae	*Sphenophryne*	*cornuta*	WNG: Wondiwoi Mts.	ZMB 62198	EU100325	EU100209
Microhylidae	Asterophryinae	*Sphenophryne*	*cornuta*	WNG: Wondiwoi Mts.	ZMB 70309	EU100326	EU100210
**Microhylidae**	**Asterophryinae**	***Xenobatrachus***	***sp. 1***	**PNG: Gulf: Sobo**	**LSUMZ 94840**	**JN049005**	**JN048980**
**Microhylidae**	**Asterophryinae**	***Xenobatrachus***	***sp. 1***	**PNG: Gulf: Sobo**	**LSUMZ 94835**	**JN049024**	**JN048999**
**Microhylidae**	**Asterophryinae**	***Xenobatrachus***	***sp. 2***	**PNG: Gulf: Sobo**	**LSUMZ 95999**	**JN049009**	**JN048984**
**Microhylidae**	**Asterophryinae**	***Xenobatrachus***	***sp. 3***	**PNG: Sandaun: Utai**	**LSUMZ 94828**	**JN049012**	**JN048987**
**Microhylidae**	**Asterophryinae**	***Xenobatrachus***	***sp. 4***	**PNG: Sandaun: Utai**	**LSUMZ94829**	**JN049013**	**JN048988**
Microhylidae	Asterophryinae	*Xenorhina*	*bouwensi*	WNG: Wondiwoi Mts.	ZMB 62639	EU100327	EU100211
Microhylidae	Asterophryinae	*Xenorhina*	*bouwensi*	WNG: Wondiwoi Mts.	ZMB 62638	EU100328	EU100212
Microhylidae	Asterophryinae	*Xenorhina*	*bouwensi*	WNG: Wondiwoi Mts.	ZMB 65138	EU100329	EU100213
Microhylidae	Asterophryinae	*Xenorhina*	*bouwensi*	WNG: Wondiwoi Mts.	ZMB 65139	EU100330	EU100214
Microhylidae	Asterophryinae	*Xenorhina*	*lanthanites*	WNG: Yapen Isl.	ZMB 69561	EU100331	EU100215
Microhylidae	Asterophryinae	*Xenorhina*	*oxycephala*	WNG: Wondiwoi Mts.	ZMB 69562	EU100332	EU100216
Microhylidae	Asterophryinae	*Xenorhina*	*varia*	WNG: Yapen Isl.	ZMB 65133	EU100333	EU100217
Microhylidae	Asterophryinae	*Xenorhina*	*varia*	WNG: Yapen Isl.	ZMB 65136	EU100334	EU100218
Microhylidae	Asterophryinae	*Xenorhina*	*varia*	WNG: Yapen Isl.	ZMB 65137	EU100335	EU100219
Microhylidae	Cophylinae	*Platypelis*	*grandis*	Madagascar: Antsiranana	AMNH A167214	DQ283410	DQ283410
Microhylidae	Cophylinae	*Platypelis*	*grandis*	Madagascar: Montagne d'Ambre	UADBA24118	EU341099	EU341099
Microhylidae	Cophylinae	*Plethodontohyla*	*bipunctata*	Madagascar: Andohahela	ZSM 89/2004	EU341068	EU341068
Microhylidae	Cophylinae	*Plethodontohyla*	*sp.*	Madagascar: Antsiranana	AMNH A167315	DQ283409	DQ283409
Microhylidae	Cophylinae	*Stumpffia*	*gimmeli*	Madagascar: Berara	ZSM 412/2000	EU341084	EU341084
Microhylidae	Cophylinae	*Stumpffia*	*psologlossa*	Madagascar: Antsiranana	AMNH A167359	DQ283411	DQ283411
Microhylidae	Dyscophinae	*Dyscophus*	*antongilii*	Madagascar: Maroantsetra	DA2005-23	EU341120	EU341120
Microhylidae	Dyscophinae	*Dyscophus*	*guineti*	pet trade	RdS	DQ283434	DQ283434
Microhylidae	Gastrophryninae	*Ctenophryne*	*geayi*	Guyana: Berbice River	AMNH A166444	DQ283383	DQ283383
Microhylidae	Gastrophryninae	*Dasypops*	*schirchi*	Brazil: Reserva do Vale	CFBH-T 71	DQ283095	DQ283095
Microhylidae	Gastrophryninae	*Elachistocleis*	*ovalis*	Guyana: Berbice River	AMNH A141136	DQ283405	DQ283405

Microhylidae	Gastrophryninae	*Gastrophryne*	*elegans*	Belize: Cockscomb Basin	RdS726	DQ283426	DQ283426
Microhylidae	Gastrophryninae	*Gastrophryne*	*olivacea*	USA: Arizona: Santa Cruz Co.	ATH 476	DQ283268	DQ283268
Microhylidae	Gastrophryninae	*Hamptophryne*	*boliviana*	Peru	RdS	DQ283438	DQ283438
Microhylidae	Hoplophryninae	*Hoplophryne*	*rogersi*	Tanzania: East Usumbaras Mts	RdS 949	DQ283419	DQ283419
Microhylidae	Kalophryninae	*Kalophrynus*	*pleurostigma*	Malaysia: Sabah	CMNH-RMB2252	DQ283146	DQ283146
Microhylidae	Microhylinae	*Calluella*	*guttulata*	Vietnam: Gia Lai Province	FMNH 252955	DQ283144	DQ283144
Microhylidae	Microhylinae	*Kaloula*	*conjuncta*	Philippines: Negros	CMNH-RMB2252	AY326064	AY326064
Microhylidae	Microhylinae	*Kaloula*	*pulchra*	Vietnam: Ha Tinh Province	AMCC 106697	DQ283397	DQ283397
Microhylidae	Microhylinae	*Kaloula*	*pulchra*	pet trade	RdS 02	DQ283398	DQ283398
Microhylidae	Microhylinae	*Microhyla*	*heymonsi*	Vietnam: Ha Giang, Yen Minh	AMNH A163850	DQ283382	DQ283382
Microhylidae	Microhylinae	*Microhyla*	*sp*	pet trade	RdS 05	DQ283422	DQ283422
Microhylidae	Phrynomerinae	*Phrynomantis*	*bifasciatus*	pet trade	RdS	DQ283154	DQ283154
Microhylidae	Phrynomerinae	*Phrynomantis*	*sp.*	—	TNHC 61077	AY326065	AY326065
Microhylidae	Scaphiophryninae	*Paradoxophyla*	*palmata*	Madagascar: Ranomafana	FGMV 2002.467	EU341121	EU341121
Microhylidae	Scaphiophryninae	*Scaphiophryne*	*brevis*	Madagascar: Berenty	MVTIS 2002-B2	EU341117	EU341117
Microhylidae	Scaphiophryninae	*Scaphiophryne*	*marmorata*	Madagascar: Antsiranana	AMNH A167395	AY843751	AY843751
